# No Stone Left Unturned: Pediatric Pancreatic Stones Presenting With Obstructive Jaundice

**DOI:** 10.1097/PG9.0000000000000217

**Published:** 2022-06-21

**Authors:** Jonathan M. deVries, Sarah Sidhu, Kathryn M. Kimsey, Grafton S. Barnett, Michael Wilsey

**Affiliations:** Department of Pediatric Gastroenterology and Nutrition, Johns Hopkins All Children’s Hospital, St. Petersburg, Florida

**Keywords:** pancreatic stones, jaundice, pancreatitis, cystic fibrosis

## Abstract

Pancreatic lithiasis, the formation of calcifications in the pancreatic duct, occurs uncommonly in pediatric patients but can occur more frequently with chronic pancreatitis (CP). Cystic fibrosis (CF) is one of the major causes of pancreatic lithiasis in pediatric patients, with mutations in the CF transmembrane conductance regulator (CFTR) gene reported in up to 23% of pediatric CP patients. Mutations in the CFTR gene can lead to mild cases of CF, which may delay diagnosis and treatment. In such cases, pancreatitis can be the presenting symptom in children with CF. We report a unique case of a 10-year-old female with previously undiagnosed and untreated CF presenting with abdominal pain, vomiting, and obstructive jaundice. Her pancreatic lithiasis and biliary obstruction were successfully treated with endoscopic retrograde cholangiopancreatography (ERCP).

## INTRODUCTION

Pancreatic lithiasis, also known as calcifying pancreatitis, is characterized by the formation of stones in the pancreatic duct or its branches. Ductal obstruction can lead to destruction and atrophy of the pancreatic parenchyma, and patients typically present with abdominal pain and emesis (1). However, obstructive jaundice in the absence of biliary abnormalities is uncommon (2). Pancreatic stone formation is related to chronic pancreatitis (CP). Although there are many causes of pancreatitis in children, genetic mutations are among the most common etiologies, with cystic fibrosis (CF) being one of the more frequently identified genetic diseases. CF is a heterogeneous disease with significant variability in organ-specific clinical manifestations (3). Pancreatitis can be the presenting symptom in children with CF (3). In a multicenter study of 155 patients, mutations in the CF transmembrane conductance regulator (CFTR) gene were reported in 34% and 23% of acute recurrent pancreatitis and chronic pediatric pancreatitis patients, respectively (4). Many mutations in the CFTR gene can lead to mild cases of CF, which may delay diagnosis and treatment (3,5). We report a unique case of a 10-year-old female with previously undiagnosed and untreated CF presenting with abdominal pain, vomiting, and obstructive jaundice. Her pancreatic stones and biliary obstruction were successfully treated with endoscopic retrograde cholangiopancreatography (ERCP).

## CASE REPORT

A previously healthy 10-year-old female with no history of pancreatitis presented to the emergency department dehydrated and mildly cachectic after 4 days of acute right upper quadrant abdominal pain, emesis, and new-onset jaundice. There was no history of previous respiratory symptoms, including sinusitis, bronchitis, or chronic cough, and the patient did not endorse diarrhea. No family history of CF was noted, but the patient’s guardian reports the newborn screen may have been positive for CF. However, due to a complex social situation, the patient moved to another state and did not receive further diagnostic evaluation nor treatment for CF.

At presentation, the patient had a weight of 28.6 kg (19th percentile; z score –0.84), height of 143 cm (74th percentile; z score 0.63), and a body mass index (BMI) of 14 kg/m^2^ (4.5th percentile; z score –1.69). Physical examination was remarkable for jaundice and right upper quadrant abdominal pain. No digital clubbing noted. Laboratory evaluation revealed total bilirubin 1.8 mg/dL (<0.8 mg/dL), direct bilirubin 1.03 mg/dL (<0.3 mg/dL), γ-glutamyl transpeptidase 250 U/L (<30 U/L), lipase 343 U/L (<39 U/L), aspartate aminotransferase 276 U/L (<26 U/L), alanine transaminase 522 U/L (<22 U/L).

**FIGURE 1.**
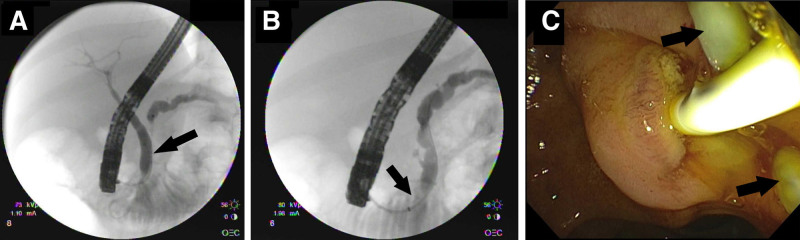
A) Cholangiogram during ERCP reveals a dilated biliary tree with no evidence of choledocholithiasis or strictures to explain the biliary obstruction (arrow indicating common bile duct). B) Fluoroscopic image showing intraductal pancreatic lithiasis (arrow indicating radiopaque stones). C) Balloon sweeping of the pancreatic duct during ERCP with removal of pancreatic lithiasis (arrows indicating pancreatic stones). ERCP = endoscopic retrograde cholangiopancreatography.

An abdominal computer tomography (CT) scan showed extrahepatic biliary dilation, with the common bile duct dilated to 7 mm and the pancreatic duct dilated to 8.8 mm. Abdominal magnetic resonance imaging (MRI) confirmed the CT findings. No definitive mass was seen. A linear filling defect of 1.4 cm × 0.3 cm was observed in the pancreatic duct, and there was no evidence of gallstones. ERCP revealed a dilated biliary tree with no stones or strictures (Fig. 1A). There were several pancreatic intraductal filling defects noted (Fig. 1B). Pancreatic and biliary sphincterotomies were performed using a Dreamtome RX cannulating sphincterotome (Boston Scientific, Marlborough, MA) and electrocautery. The biliary and pancreatic ducts were swept with a 9–12 mm Extractor Pro RX Retrieval Balloon Catheter (Boston Scientific, Marlborough, MA), and 6 white pancreatic stones were successfully removed from the pancreatic duct (Fig. 1C). A 5 Fr × 7 cm prophylactic pigtail pancreatic stent (Cook Medical, Bloomington, IN) was placed.

Following the procedure, genetic testing revealed heterozygous F508del and 11TG-5T mutations in the CFTR gene. However, sweat chloride was normal at 17 mmol/L (<40 mmol/L). Although the patient did not endorse diarrhea or steatorrhea, the stool elastase was low at <15 µg/g stool (normal > 200 µg/g), suggesting exocrine pancreatic insufficiency. She was prescribed pancreatic enzyme replacement therapy and her weight had increased by 3.44 kg at her 1-month follow-up visit (BMI 15.7 kg/m^2^; 27th percentile; z score –0.61).

## DISCUSSION

Obstructive jaundice is an atypical presentation of pancreatic stones in children (1). Acute biliary obstruction occurs when a pancreatic stone obstructs the common channel (the distal junction of the common bile duct and pancreatic duct entering the duodenum). Pancreatic stones are common in adults, occurring in up to 60% of all patients with CP and up to 90% of patients with alcohol-related pancreatitis (1,6). Although pancreatic stones are uncommon in children, they can occur in the setting of CP. In one study of 75 pediatric patients with idiopathic CP in China, 45 (60%) had evidence of pancreatic ductal stones (7).

Previous studies have noted that CF-related pancreatitis is more common in patients with milder cases of CF, including those with pancreatic sufficiency (8). Pancreatic insufficient individuals, while making up a greater proportion of the CF population, present less frequently with pancreatitis (8). Our patient’s complex social situation, combined with a double heterozygote CFTR gene mutation reflecting a milder CF course, likely contributed to her delayed diagnosis and treatment.

Currently, ultrasound, CT, and MRI are used to identify pancreatic stones, but ERCP is often required for removing stones and relieving ductal obstruction (9). ERCP can be limited by larger stone size or by strictures within the pancreatic ducts. In such cases, extracorporeal shock wave lithotripsy has been shown effective at fragmenting the stones for subsequent removal by ERCP (6).

Newer therapies, such as CFTR modulators, have shown great promise in treating CFTR-related pancreatic disease (10). However, future studies are needed to determine whether CFTR modulator treatment may provide risk-reduction of pancreatic ductal stone formation in the setting of CFTR-related CP.

We report a unique case of a 10-year-old female with previously undiagnosed and untreated CF presenting with obstructive jaundice. Her pancreatic stones and biliary obstruction were successfully treated with ERCP.

## ACKNOWLEDGMENTS

This statement serves to confirm that informed patient consent was obtained from the patient’s legal guardian for publication of the case details.
